# Supplementary material for: Theoretical Study on the Origin of Abnormal Regioselectivity in Ring-Opening Reaction of Hexafluoropropylene Oxide
Source: Molecules. 2023 Feb 9;28(4):1669. doi: 10.3390/molecules28041669 (PMC9962681; doi:10.3390/molecules28041669)
Supplement: Supplementary file 1 [file molecules-28-01669-s001.zip › molecules-2117507-supplementary.pdf]

SUPPLEMENTARY INFORMATION

Theoretical Study on the Origin of Abnormal Regioselectivity in Ring-opening Reaction of Hexafluoropropylene Oxide

Cui Yu [1], Yueqian Sang [1], Yao Li [1,*] and Xiaosong Xue [1,2,*]

1 Key Laboratory of Organofluorine Chemistry, Shanghai Institute of Organic Chemistry, University of Chinese Academy of Sciences, Chinese Academy of Sciences, 345 Lingling Road, Shanghai, 200032, China

2 School of Chemistry and Materials Science, Hangzhou Institute for Advanced Study, University of Chinese Academy of Sciences, 1 Sub-lane Xiangshan, Hangzhou 310024, China

* Correspondence: liyao@sioc.ac.cn (Y.L.); xuexs@sioc.ac.cn (X.X.)

Contents

Part 1. Negative hyperconjugation in **TS1α** and **TS1β**. S2

Part 2. The reactivity descriptors of epoxide carbon atoms of **1** and **2**. S2

Part 3. Key NBOs of **1** and **2.** S3

Part 4. Reference. S3

Part 5. ωB97X-D/6-31+G(d,p)-SMD-(ethyl ether) Calculated Cartesian Coordinates and Single Points Energy Calculated Using the ωB97X-D/6-311++G(2df,2p)-SMD-(ethyl ether).S4

Part 6. ωB97X-D/6-31G(d,p)-SMD-(ethyl ether) Calculated Cartesian Coordinates...........S8

Part 1. Negative hyperconjugation in TS1α and TS1β.

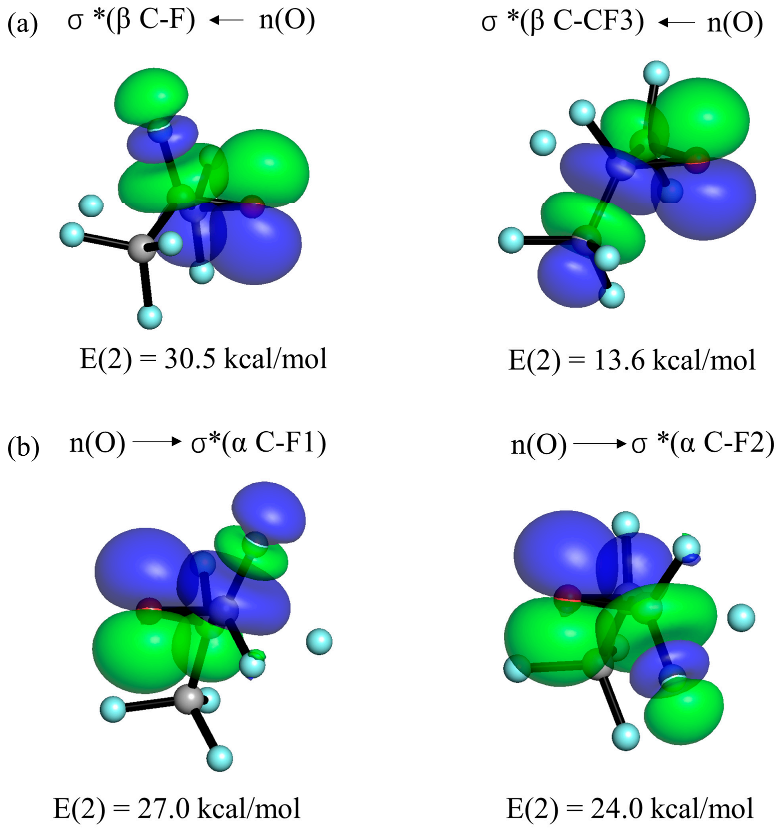

Figure S1. The key donor-acceptor orbital interactions in **TS1α**(a) and **TS1β**(b) by NBO analysis at the ωB97X-D/6-31+G(d,p)-SMD-(ethyl ether) level.

Part 2. The reactivity descriptors of epoxide carbon atoms of 1 and 2.

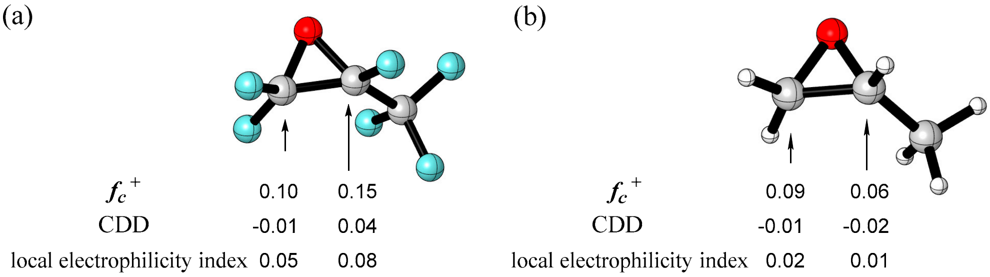

Figure S2. The reactivity descriptors of epoxide carbon atoms of **1**(a) and **2**(b), respectively.

To investigate the influence of electrophilicity on the regioselectivity, we conducted conceptual density functional theory (CDFT) [1-4] calculation (Figure S2). Interestingly, condensed Fukui function f_c^+ [5,6], condensed dual descriptors CDD [7], and condensed local electrophilicity index [8] all indicate that β-C should be more inclined to be attacked. These results remind us to visualize the lowest unoccupied molecular orbital and key Natural Bond Orbitals (NBOs) of **1** and **2**.

Part 3. Key NBOs of 1 and 2.

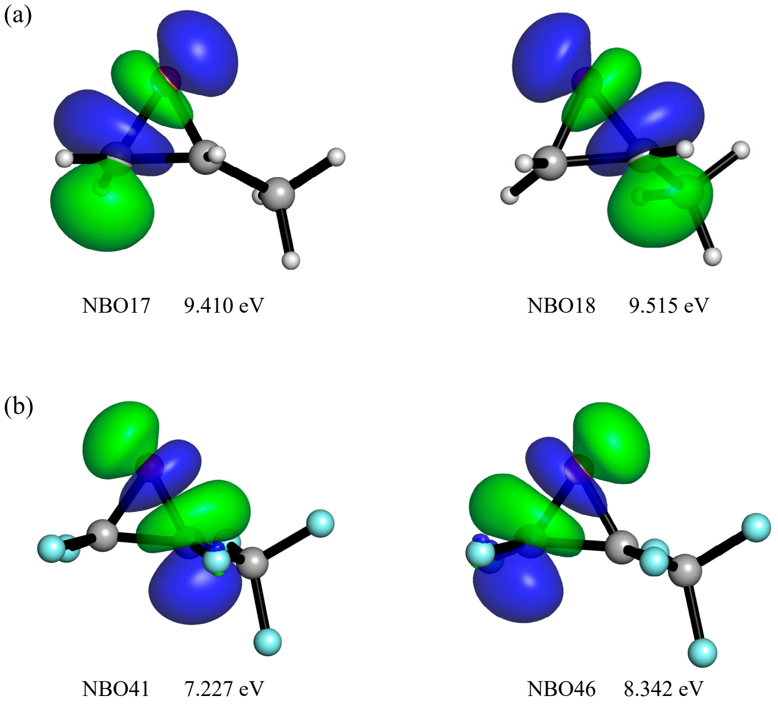

Figure S3. The key NBOs isosurfaces of **2**(a) and **1**(b) obtained at the ωB97X-D/6-31G(d,p)-SMD-(ethyl ether). Orbital energies are provided below the isosurfaces

NBO analysis shows that the energy of $\sigma^*_{C(\beta)-O}$ is lower than that of $\sigma^*_{C(\alpha)-O}$ for **1**, while the opposite trend was observed for **2** (Figure S2). These results indicate that charge-transfer or donor-acceptor orbital interactions between the incoming fluoride and the antibonding orbital of C-O bond in **1**/**2** may contribute to both the reactivity and regioselectivity.

Part 4. Reference.

1. Geerlings, P.; De Proft, F.; Langenaeker, W. Conceptual density functional theory. *Chem. Rev.* **2003**, *103*, 1793-873.
2. Shu-Bin, L. I. U. Conceptual Density Functional Theory and Some Recent Developments. *Acta. Phys. Chim. Sin.* **2009**, *25*, 590-600.
3. Rong, F. U.; Tian, L. U.; Fei-Wu, C. Comparing Methods for Predicting the Reactive Site of Electrophilic Substitution. *Acta. Phys. Chim. Sin.* **2014**, *30*, 628-639.
4. Domingo, L. R.; Rios-Gutierrez, M.; Perez, P. Applications of the Conceptual Density Functional Theory Indices to Organic Chemistry Reactivity. *Molecules* **2016**, *21*, 748.
5. Parr, R. G.; Yang, W. Density functional approach to the frontier-electron theory of chemical reactivity. *J. Am. Chem. Soc.* **1984**, *106*, 4049-4050.
6. Yang, W.; Mortier, W. J. The use of global and local molecular parameters for the analysis of the gas-phase basicity of amines. *J. Am. Chem. Soc.* **1986**, *108*, 5708-5711
7. Morell, C.; Grand, A.; Toro-Labbe, A. New dual descriptor for chemical reactivity. *J. Phys. Chem. A* **2005**, *109*, 205-212.
8. Parr, R. G.; Szentpály, L. V.; Liu, S. Electrophilicity Index. *J. Am. Chem. Soc.* **1999**, *121*, 1922-1924.

Part 5. ωB97X-D/6-31+G(d,p)-SMD-(ethyl ether) Calculated Cartesian Coordinates and Single Points Energy Calculated Using the ωB97X-D/6-311++G(2df,2p)-SMD-(ethyl ether).

1

C	1.40388	0.17884	0.00822
C	0.11485	-0.49349	0.07616
O	0.87325	-0.17061	1.22632
F	1.56207	1.47411	-0.22893
C	-1.26128	0.16294	-0.05531
F	0.04058	-1.79959	-0.2125
F	-1.69058	0.06395	-1.31633
F	-1.21135	1.44976	0.28355
F	-2.13735	-0.4545	0.74261
F	2.48878	-0.48094	-0.37785

Zero-point correction= 0.038925 (Hartree/Particle)
Thermal correction to Energy= 0.047239
Thermal correction to Enthalpy= 0.048183
Thermal correction to Gibbs Free Energy= 0.004663
SCF Done: E(RwB97XD) = -788.611652881

2

C	1.0376	0.61743	-0.05631
C	-0.15168	-0.04215	0.48702
O	0.82787	-0.78509	-0.24634
C	-1.50612	0.09917	-0.14851
H	1.86718	0.87513	0.59986
H	-0.15264	-0.27027	1.5534
H	-2.07022	0.89959	0.34203
H	-2.07678	-0.82967	-0.04766
H	-1.41436	0.33741	-1.21233
H	0.94507	1.22184	-0.95786

Zero-point correction= 0.086283 (Hartree/Particle)
Thermal correction to Energy= 0.090652
Thermal correction to Enthalpy= 0.091597
Thermal correction to Gibbs Free Energy= 0.059939
SCF Done: E(RwB97XD) = -193.120552207

Int1α

C	1.30219	-0.19929	-0.00229
C	0.0001	0.64047	-0.25307
O	0.00173	1.2995	-1.31618
F	2.38414	0.59623	-0.03643
F	1.34367	-0.86685	1.17172
C	-1.30277	-0.19878	-0.00261
F	1.46858	-1.1286	-0.97003
F	-1.42516	-1.18031	-0.9263
F	-1.38239	-0.80311	1.20204
F	-2.38909	0.58258	-0.11774
F	-0.00097	1.48335	1.01866

Zero-point correction= 0.040002 (Hartree/Particle)
Thermal correction to Energy= 0.049801

Thermal correction to Enthalpy= 0.050745
Thermal correction to Gibbs Free Energy= 0.003517
SCF Done: E(RwB97XD) = -888.652430169

Int1β

C	0.05136	0.5408	-0.00325
C	1.32133	-0.35267	0.18258
O	1.36119	-1.09981	1.1584
C	-1.29829	-0.22337	0.00624
F	-1.32711	-1.18908	-0.91772
F	0.07662	1.25945	-1.15801
F	1.47255	-0.99817	-1.11454
F	2.39685	0.63025	0.0898
F	-1.55435	-0.7684	1.197
F	-2.30416	0.62993	-0.26941
F	-0.01995	1.43712	1.01947

Zero-point correction= 0.040180 (Hartree/Particle)
Thermal correction to Energy= 0.049851
Thermal correction to Enthalpy= 0.050795
Thermal correction to Gibbs Free Energy= 0.004763
SCF Done: E(RwB97XD) = -888.657861420

Int2α

C	-0.77042	-0.61991	0.25361
C	0.55753	-0.12251	-0.32972
O	1.52689	-0.99047	0.03806
F	-1.90534	0.17979	-0.12362
C	0.81115	1.33177	0.12237
H	-0.73877	-0.60234	1.34906
H	-0.9847	-1.6321	-0.10074
H	0.91345	1.36618	1.21596
H	0.39362	-0.0685	-1.44281
H	0.00722	2.01918	-0.17755
H	1.75261	1.68709	-0.31333

Zero-point correction= 0.087433 (Hartree/Particle)
Thermal correction to Energy= 0.093003
Thermal correction to Enthalpy= 0.093947
Thermal correction to Gibbs Free Energy= 0.058764
SCF Done: E(RwB97XD) = -293.079702585

Int2β

C	-0.39466	-0.04831	-0.34087
C	0.93175	-0.55416	0.25468
O	1.98812	0.18294	-0.14124
C	-0.79184	1.33018	0.12517
F	-1.4604	-0.94947	0.0284
H	-0.34681	-0.0995	-1.43483
H	0.98026	-1.64109	-0.03411
H	0.76184	-0.57425	1.37003
H	0.0072	2.02752	-0.1418
H	-1.72888	1.66194	-0.33482
H	-0.90649	1.3408	1.21594

Zero-point correction= 0.087079 (Hartree/Particle)
Thermal correction to Energy= 0.092672
Thermal correction to Enthalpy= 0.093616
Thermal correction to Gibbs Free Energy= 0.058370
SCF Done: E(RwB97XD) = -293.080411305

P1α

C	-1.32702	-0.09815	-0.00399
C	-0.00001	0.71231	-0.00008
O	-0.00002	1.90592	-0.00001
F	-2.32897	0.66662	-0.42043
F	-1.23493	-1.1661	-0.80416
C	1.32705	-0.09808	0.00393
F	-1.59497	-0.51959	1.23871
F	1.23518	-1.16546	0.80504
F	1.59452	-0.5206	-1.23846
F	2.32917	0.66692	0.4194

Zero-point correction= 0.038593 (Hartree/Particle)
Thermal correction to Energy= 0.047442
Thermal correction to Enthalpy= 0.048386
Thermal correction to Gibbs Free Energy= 0.003142
SCF Done: E(RwB97XD) = -788.641983264

P1β

C	0.168	0.49094	-0.33066
C	1.4986	-0.0975	0.1941
O	2.1452	0.34282	1.07826
C	-1.08845	-0.25548	0.20454
F	-1.09665	-1.51744	-0.22515
F	0.14979	0.44284	-1.6765
F	1.83024	-1.19221	-0.48787
F	-1.07416	-0.25466	1.53943
F	-2.19102	0.35466	-0.21922
F	0.08953	1.77011	0.06553

Zero-point correction= 0.038748 (Hartree/Particle)
Thermal correction to Energy= 0.047580
Thermal correction to Enthalpy= 0.048524
Thermal correction to Gibbs Free Energy= 0.003528
SCF Done: E(RwB97XD) = -788.651092777

ts1α

C	1.26191	0.04834	0.25973
C	-0.03973	0.59714	-0.04394
O	0.39061	1.20642	1.07646
F	1.47925	-0.89969	1.13741
F	1.4049	-1.2998	-1.24406
F	2.35624	0.64936	-0.13749
C	-1.3605	-0.18505	0.04772
F	-2.34395	0.66415	0.40868
F	-1.71467	-0.74316	-1.11165
F	-1.2994	-1.14637	0.97637
F	-0.13736	1.39617	-1.16179

Zero-point correction= 0.038582 (Hartree/Particle)
Thermal correction to Energy= 0.048337
Thermal correction to Enthalpy= 0.049281
Thermal correction to Gibbs Free Energy= 0.002888
SCF Done: E(RwB97XD) = -888.571642171

ts1β

C	-0.12305	0.29547	-0.35667
C	-1.39163	-0.31221	-0.02996
O	-0.89216	-0.95456	-1.08226
C	1.27142	-0.22996	0.02768
F	1.26835	-0.85591	1.20391
F	0.00587	1.60717	1.2191
F	-1.58515	-1.03624	1.0945
F	-2.53061	0.39372	-0.2286
F	1.70356	-1.12148	-0.89204
F	2.17921	0.74483	0.04429
F	-0.08603	1.28088	-1.23986

Zero-point correction= 0.038559 (Hartree/Particle)
Thermal correction to Energy= 0.048248
Thermal correction to Enthalpy= 0.049192
Thermal correction to Gibbs Free Energy= 0.003092
SCF Done: E(RwB97XD) = -888.577972634

ts2α

C	-0.54487	-0.68452	0.1854
C	0.61662	-0.03415	-0.41703
O	1.27573	-1.13961	0.09282
F	-2.13719	0.30156	-0.04825
C	1.03209	1.31767	0.13766
H	-0.63694	-0.62602	1.2607
H	-0.96192	-1.54193	-0.322
H	1.01422	1.30053	1.23371
H	0.58002	0.00853	-1.5207
H	0.35795	2.1092	-0.21152
H	2.05246	1.55856	-0.18469

Zero-point correction= 0.085834 (Hartree/Particle)
Thermal correction to Energy= 0.091537
Thermal correction to Enthalpy= 0.092482
Thermal correction to Gibbs Free Energy= 0.056866
SCF Done: E(RwB97XD) = -293.056455571

ts2β

C	-0.08624	0.04428	-0.33405
C	0.88842	-0.78284	0.36831
O	1.82251	0.03525	-0.24135
C	-0.46494	1.40167	0.17534
F	-1.83011	-0.75343	-0.04282
H	-0.17843	-0.14667	-1.3945
H	0.9032	-1.84651	0.07278
H	0.83468	-0.71859	1.47074
H	0.26096	2.1505	-0.15057

H	-1.44915	1.67672	-0.20923
H	-0.50383	1.40474	1.26936

Zero-point correction= 0.085744 (Hartree/Particle)
Thermal correction to Energy= 0.091572
Thermal correction to Enthalpy= 0.092516
Thermal correction to Gibbs Free Energy= 0.056612
SCF Done: E(RwB97XD) = -293.051506522

Part 6. ωB97X-D/6-31G(d,p)-SMD-(ethyl ether) Calculated Cartesian Coordinates.

1

C	1.39450800	0.17849400	-0.00914100
C	0.11298500	-0.50077200	-0.07484000
O	0.86780100	-0.17256600	-1.22990700
F	2.48054700	-0.47357900	0.37730000
F	1.53582600	1.47261900	0.23091400
F	0.04072100	-1.80337900	0.21547900
C	-1.25267600	0.16244000	0.05467300
F	-1.18743200	1.44686400	-0.28381000
F	-1.67984000	0.06601700	1.31436600
F	-2.13107900	-0.44859200	-0.74146000

Zero-point correction= 0.039654 (Hartree/Particle)
Thermal correction to Energy= 0.047903
Thermal correction to Enthalpy= 0.048848
Thermal correction to Gibbs Free Energy= 0.005484

2

C	1.03800900	0.61429900	-0.05658000
C	-0.15028200	-0.04521300	0.48789000
O	0.82516200	-0.78312200	-0.24777400
C	-1.50442100	0.10056200	-0.14787800
H	1.86604000	0.87652200	0.60097800
H	-0.15342700	-0.26897800	1.55596100
H	-2.07054200	0.90015100	0.34045000
H	-2.07751700	-0.82683100	-0.05200800
H	-1.40882900	0.33900600	-1.21090400
H	0.94314400	1.22721700	-0.95287700

Zero-point correction= 0.086484 (Hartree/Particle)
Thermal correction to Energy= 0.090846
Thermal correction to Enthalpy= 0.091790
Thermal correction to Gibbs Free Energy= 0.060143
